# Supplementary material for: Assessment of four in vitro phenotypic biofilm detection methods in relation to antimicrobial resistance in aerobic clinical bacterial isolates
Source: PLoS One. 2023 Nov 22;18(11):e0294646. doi: 10.1371/journal.pone.0294646 (PMC10664881; doi:10.1371/journal.pone.0294646)
Supplement: Attachment — Submitted filename: Response to Reviewers.docx [file pone.0294646.s003.docx]

**Dear editor,**

Thank you for giving us the opportunity to submit a revised draft of our manuscript titled “**Assessment of four *in vitro* phenotypic biofilm detection methods in relation to antimicrobial resistance in aerobic clinical bacterial isolates”** to PLOS ONE. We appreciate the time and effort that you and the four reviewers have dedicated to providing valuable feedback on our manuscript. We are grateful to the reviewers for their insightful comments on this study. We have been able to incorporate changes to reflect most of the suggestions provided by the reviewers. Please find enclosed our response to the reviewer’s comments.

| **Date:** | Mar 30 2023 |
| --- | --- |
| **To:** | "Basanta Tamang" [tamangbasanta222@gmail.com](mailto:tamangbasanta222@gmail.com) |
| **From:** | "PLOS ONE" plosone@plos.org |
| **Subject:** | PLOS ONE Decision: Revision required [PONE-D-23-02752] - [EMID:17d8c5f0f5e971e8] |
| **Attachment(s):** | PONE-D-23-02752_reviewer AND 2nd assignment-PONE-D-23-02752 |

PONE-D-23-02752

**Assessment of four *in vitro* phenotypic biofilm detection methods in relation to antimicrobial resistance in aerobic clinical bacterial isolates**

Dear Dr. Tamang,

Thank you for submitting your manuscript to PLOS ONE. After careful consideration, we feel that it has merit but does not fully meet PLOS ONE’s publication criteria as it currently stands. Therefore, we invite you to submit a revised version of the manuscript that addresses the points raised during the review process.

**General comments (Journal requirements)**

1. Please ensure that your manuscript meets PLOS ONE's style requirements, including those for file naming. The PLOS ONE style templates can be found at

https://journals.plos.org/plosone/s/file?id=wjVg/PLOSOne_formatting_sample_main_body.pdf and

https://journals.plos.org/plosone/s/file?id=ba62/PLOSOne_formatting_sample_title_authors_affiliations.pdf

**Response:** We thank you for this useful suggestion. We followed the links and made the necessary changes to the manuscript. As suggested, upon improvements, we have prepared and named three different files as 'Response to Reviewers' (for rebuttal letter), and ' Manuscript with Track Changes’ (for a marked-up copy of the manuscript), and ‘Revised Manuscript’ (for revised paper). Additionally, we have checked and attest that all formatting and style requirements have been met in the manuscript.

2. Please upload a completed version of your questionnaire as Supporting Information when you resubmit your manuscript.

**Response:** We have uploaded a completed version of questionnaire as “Supporting Information”.

3. Please ensure that you have an ORCID iD and that it is validated in Editorial Manager.

**Response:** Validated ORCID iD has been added.

4. Please include your full ethics statement in the ‘Methods’ section of your manuscript file. In your statement, please include the full name of the IRB or ethics committee who approved or waived your study, as well as whether or not you obtained informed written or verbal consent. If consent was waived for your study, please include this information in your statement as well.

**Response:** We have now added a full ethics statement in the ‘Methods’ section of the manuscript file, as well as a sentence concerning the written informed consent.

**Response to Reviewer 1**

1. Author should clearly mention the aim of the study in abstract and in main manuscript. Presently, the aim defined at two sites is different from each other.

**Response:** First of all, we will like to express our sincere gratitude for your kind suggestions. We appreciate the time and effort that you dedicated to providing feedback on our manuscript and are grateful for the insightful comments on our paper.

Thank you for pointing this out. We agree with this comment. Therefore, following the reviewer suggestions, the aim of the study throughout the manuscript (abstract and main text) has been updated as ... to evaluate diagnostic parameters of four *in vitro* phenotypic biofilm detection assays in relation to antimicrobial resistance in aerobic clinical bacterial isolates...

2. How was the susceptibility done for colistin and vancomycin? As per CLSI guidelines, Kirby Bauer method can't be used for these drugs.

**Response:** Thank you for pointing this out. We have now studied the CLSI guideline (32^th^ edition), and as is explicitly stated in it, we have as of this this time amend this error by erasing the susceptibility testing for colistin and vancomycin. Further, we will implement this information in routine diagnosis too.

3. Total is not 194. It is 205. Please confirm.

**Response:** Dear reviewer, there were 194 samples that tested culture positive. Of these 162 samples yielded monobacterial growth, while 32 samples yielded 2 bacterial growth

162 samples	=	162 isolates

32 samples	=	64 isolates

---------------------------------------------------------------

194 total samples	=	226 total isolates

Therefore, we confirm total samples as 194.

4. Expand ACB.

**Response:** Thank you for pointing this error. We have expanded the abbreviation accordingly. … *Acinetobacter calcoaceticus-baumanii* (ACB) complex….

5. What is the relevance of calculating p-value here in this table?

**Response:** Thank you for this comment. Actually, the table shows interesting and significant association of pathogen with the samples. For e.g., as we know, *E. coli* as a significant uropathogen, *S.* Typhi as a significant bloodstream pathogen, non-fermenters as the significant respiratory pathogens, and lastly *S. aureus* as the significant wound pathogen; all of these routine findings are explicitly portrayed in the table (significant correlation as per p-value reiterates the preexisting fact).

6. How do you differentiate between colonizers and pathogens?

**Response:** As we know, colonization is the presence of bacteria on a body surface (like on the skin, mouth, intestines or airway) without causing disease in the person. Infection is the invasion of a host organism's bodily tissues by disease-causing organisms. The samples obtained in the study were stringently from hospital visiting patients clinically suspected of infection. Hence, they were most likely to be pathogens.

7. Table 6 is not needed. Author has already shown relationship between biofilm formation and drug resistance (MDR & XDR) in table 5.

**Response:** Dear Reviewer, table 5 is discrete to table 6 in context to simply portraying the pathogen specific incidences of MDR-, XDR- or biofilm formation. The latter distinctly, however, shows antibiotic specific incidences of resistance (%) to each of the tested, be it with regard to MDR-, XDR-, or biofilm-MDR-, or biofilm-XDR-isolates. Furthermore, table 6 summarizes the overall resistance to each antibiotic tested, while table 5 fundamentally shows the frequencies. Therefore, we sincerely request you to reconsider the urgency of table 6.

**Response to Reviewer 2**

1. All the revisions and very important notes are documented on the text. To follow and track and complete your manuscript. It is need to update the references and adding as we mentioned in the text

**Response:** First of all, we will like to express our sincere gratitude for your kind suggestions. We appreciate the time and effort that you dedicated to providing feedback on our manuscript and are grateful for the insightful comments on our paper.

Thank you for your kind suggestions. We have now updated the title as “**Assessment of four *in vitro* phenotypic biofilm detection methods in relation to antimicrobial resistance in aerobic clinical bacterial isolates**” We have added all the mentioned references in the study as well as incorporated the indicated change(s).
