# Supplementary material for: Assessment of four in vitro phenotypic biofilm detection methods in relation to antimicrobial resistance in aerobic clinical bacterial isolates
Source: PLoS One. 2023 Nov 22;18(11):e0294646. doi: 10.1371/journal.pone.0294646 (PMC10664881; doi:10.1371/journal.pone.0294646)
Supplement: Attachment — Submitted filename: Response to Reviewers.docx [file pone.0294646.s005.docx]

**Dear editor,**

Thank you for giving us the opportunity to submit a revised draft of our manuscript titled “**Assessment of four *in vitro* phenotypic biofilm detection methods in relation to antimicrobial resistance in aerobic clinical bacterial isolates”** to PLOS ONE. We appreciate the time and effort that you and the three reviewers have dedicated to providing valuable feedback on our manuscript. We are grateful to the reviewers for their insightful comments on this study. We have been able to incorporate changes to reflect most of the suggestions provided by the reviewers. Please find enclosed our response to the reviewer’s comments.

| **Date:** | October 13 2023 |
| --- | --- |
| **To:** | "Basanta Tamang" [tamangbasanta222@gmail.com](mailto:tamangbasanta222@gmail.com) |
| **From:** | "PLOS ONE" plosone@plos.org |
| **Subject:** | PLOS ONE Decision: Revision required [PONE-D-23-02752R1] - [EMID:2eef6614e577405d] |

PONE-D-23-02752R1

**Assessment of four *in vitro* phenotypic biofilm detection methods in relation to antimicrobial resistance in aerobic clinical bacterial isolates**

**Journal Requirements:**

**Please review your reference list to ensure that it is complete and correct. If you have cited papers that have been retracted, please include the rationale for doing so in the manuscript text, or remove these references and replace them with relevant current references. Any changes to the reference list should be mentioned in the rebuttal letter that accompanies your revised manuscript. If you need to cite a retracted article, indicate the article’s retracted status in the References list and also include a citation and full reference for the retraction notice.**

**Yes, we have made substantial changes in the reference/citations in the manuscript. Specifically, we have removed the references** 7, 8, 9, and 32 from the manuscript following the revision request. The total number of references in the current article is 37, which were 41 in the prior version.

**Editor Comments**

**"We note that one or more reviewers has recommended that you cite specific previously published works. As always, we recommend that you please review and evaluate the requested works to determine whether they are relevant and should be cited. It is not a requirement to cite these works. We appreciate your attention to this request.”**

**Response:** We are grateful to the reviewers for highlighting frequent, specific citations in this study. Revising the manuscript entirely with a particular focus on the textual context and referenced article, we abridged 5 citations/references. The curtailed manuscript definitely looks more promising than before. We thank you for this useful suggestion.

Here are the details.

Reference numbers 7, 8, and 9

We have gone through the articles *“ESCMID guideline for the diagnosis and treatment of biofilm infections 2014”, “A study on device-related infections with special reference to biofilm production and antibiotic resistance" and “Effect of pyocyanin as secondary metabolite on pseudomonal biofilm and in increasing the resistance degree to antipseudomonal agents”* and realized that

The statement (lines 106-108)…….*different bacterial species may contribute to the development of biofilms, which may open up the possibility of quorum sensing for horizontal gene transmission within the spatially well-organized architecture*…. is coherently described in reference number 4—future perspectives—justifying the basic concept of quorum sensing. We agree with the reviewers’ suggestions of adding these references 7, 8, and 9 as only the burden in the manuscript.

Therefore, we have now removed the citation and references 7, 8, and 9 accordingly from the manuscript.

Reference number 13

We realize that the sentence (lines 116-119) ……*Phenotypic methods, such as the tissue culture plate method (TCPM) (quantitative), tube method (TM) (qualitative), Congo red agar (CRA) (qualitative), and modified Congo red agar (MCRA) (qualitative), can be used to detect biofilm-forming properties in a simple, rapid, and relatively sensitive manner….* is justified by the article “*Biofilm antimicrobial susceptibility pattern for selected antimicrobial agents against planktonic and sessile cells of clinical isolates of staphylococci using MICs, BICs and MBECs*."

The article exhaustively discusses the inhibitory concentrations, particularly for inhibition and eradication of biofilms, and using the gold standard technique, i.e. Tissue Culture Plate Method, with the detailed methodology.

Therefore, we have now maintained the citation/reference 13 in the manuscript.

Reference number 32

The sentence (lines 394-398)….*This could be because we use these drugs only to treat infections with MDR bacteria as a last-line therapy in our setting, and because of their infrequent use, the emergence and dissemination of penicillins with beta-lactamase inhibitors- and aminoglycoside-resistant genes are minimal, which restrains the emergence of resistance to these antibiotics*……..is a typical finding based on our research institution. This pattern may vary between institutions as the bacteria's selection due to antimicrobial resistance mechanisms varies. Therefore, there is no need for any citations/references to support the finding.

While the article “*Ciprofloxacin-and gentamicin-mediated inhibition of Pseudomonas aeruginosa biofilms is enhanced when combined the volatile oil from Eucalyptus camaldulensis*” is specific to *P. aeruginosa* and mostly discusses the genetic mechanism and inhibitory concentrations, the article is beyond the scope of the manuscript.

Therefore, we have now removed the citation and reference accordingly from the manuscript.

Reference number 41

The latter part of the sentence (lines 474-477)….*These higher antimicrobial resistance rates among MDR- and XDR-biofilm producers may be due to the biofilm matrix's protective function, which reduces antibiotic penetration, or to plasmid-encoded genes being uptaken by bacteria via quorum sensing mechanisms*….discusses the acquisition of genes during quorum sensing process in biofilms. This is directly justified by the article “*Molecular Detection of Medically Important Carbapenemases Genes Expressed by Metallo-Î²-lactamase Producer Isolates of Pseudomonas aeruginosa and Klebsiella pneumonia*” by Al-Ouqaili et al. 2018 discussing the significance of beta-lactamases with or without the resistance genes *bla_OXA_* genes in fermenters and non-fermenters.

Therefore, we have used the reference in the article.

**Response to Reviewer 1**

1. **Reviewer #1: the author has addressed all the comments and has made appropriate changes. The author has provided the data in well-understandable manner and has included the tables appropriately.**

**Response:** Please accept our sincerest thanks for your time and consideration.

**Response to Reviewer 2**

1. **Dear Author, You are doing well. There are simple errors in writing the two references in numbers 13 and 41 in the list of references of the revised manuscript which the contributing authors are missing. Thus, the below the revisions:-
   Reference 13:- The corrected and revised form of reference 13 is as follow:-Al-Ouqaili, M.T.S., Al-Kubaisy, S.H.M., Al-Ani, N.F.I. Biofilm antimicrobial susceptibility pattern for selected antimicrobial agents against planktonic and sessile cells of clinical isolates of staphylococci using MICs, BICs, and MBECs. Asian Journal of Pharmaceutics, Volume 12, Issue 4, October-December 2018, Pages S1375-S1383 instead of the citation form in the list of references.**

**Response:** First of all, we will like to express our sincere gratitude for your kind suggestions. We appreciate the time and effort that you dedicated to providing feedback on our manuscript and are grateful for the insightful comments on our paper.

Thank you for pointing this out. We agree with this comment. Therefore, following the reviewer suggestions, we have updated the reference as:

Al-Ouqaili MTS, Al-Kubaisy SHM, Al-Ani NFI. Biofilm antimicrobial susceptibility pattern for selected antimicrobial agents against planktonic and sessile cells of clinical isolates of staphylococci using MICs, BICs and MBECs. Asian Journal of Pharmaceutics (AJP). 2018;12(04):S1375-S1383.

2. **Reference 14:- The corrected and revised form of reference 41 is as follow:-Al-Ouqaili, MTS, Al-Taei, SA, Al-Najjar A. Molecular Detection of Medically Important Carbapenemases Genes Expressed by Metallo-β-lactamase Producer Isolates of Pseudomonas aeruginosa and Klebsiella pneumoniae. Asian Journal of Pharmaceutics • Jul -Sep 2018 (Suppl ) • 12 (3) | S991 instead of the citation form in the list of references.**

**Response:** Thank you for pointing this out. We have updated the reference as follows: Al-Ouqaili MTS, Al-Taei SA, Al-Najjar A. Molecular Detection of Medically Important Carbapenemases Genes Expressed by Metallo-β-lactamase Producer Isolates of Pseudomonas aeruginosa and Klebsiella pneumoniae. Asian Journal of Pharmaceutics. Jul -Sep 2018 (Suppl); 12 (3):S991

Additionally, the authors plead the reviewer to consider limiting the two suggested references/citations based on the editor's and other reviewers' advice to *"avoid citing specific previously published works."* In light of its use, we find the manuscripts less relevant (please refer to the response for academic editor above). As a microbiology enthusiast, I (corresponding author) sincerely acknowledge the importance of those papers, especially in relation to cystic fibrosis, quorum sensing, and *pel* genes—my concurrent study. It would be my pleasure to reach out to the author and incorporate the published intellect into the ongoing paper as it will be more relevant in the paper, and request the reviewer to consider this concurrent issue of specific reference/citation.

**Response to Reviewer 3**

1. **The authors have well addressed the reviewer’s comments however there are minor clarifications in results required from authors. Table 2: why no of isolates are more than no of samples of urine blood and sputum**

**Response:** First of all, we will like to express our sincere gratitude for your kind suggestions. We appreciate the time and effort that you dedicated to providing feedback on our manuscript and are grateful for the insightful comments on our paper.

A total of 194 samples that tested culture positive in this study. Of these, 162 samples yielded monobacterial growth, while 32 samples yielded 2 bacterial growth. Kindly look into the calculation below:

162 samples	=	162 isolates	*Monobacterial*

32 samples	=	64 isolates	*Polybacteral (*2)*

---------------------------------------------------------------

194 total samples	=	226 total isolates

Therefore, there were higher numbers of bacterial isolates than the number of samples.

The fact that the number of isolates is more than number of urine, blood and sputum samples is because of isolation of higher number of bacteria. There were 101 bacteria isolated from urine (n=83), 33 from blood (n=29) and 71 from sputum samples (n=62). The following table clearly depicts the incidences of monobacterial and polybacterial in clinical samples

| **Samples** | **Mono** | **Poly (*2)** | **Total** |
| --- | --- | --- | --- |
| Blood | 25 | 4 | 33 |
| Urine | 65 | 18 | 101 |
| Sputum | 53 | 9 | 71 |
| Wound Swabs | 4 | 0 | 4 |
| Pus | 6 | 1 | 8 |
| Ascitic fluid | 5 | 0 | 5 |
| Pleural fluid | 1 | 0 | 1 |
| Catheter tips | 3 | 0 | 3 |
| Total | 162 | 32 | 226 |

2. **Table 2: what is the significance of p-value**

**Response:** Thank you for this comment. Actually, the table shows interesting and significant association of pathogen with the samples. For e.g., as we know, *E. coli* as a significant uropathogen, *S.* Typhi as a significant bloodstream pathogen, non-fermenters as the significant respiratory pathogens, and lastly *S. aureus* as the significant wound pathogen; all of these routine findings are explicitly (strongly) portrayed in the table (significant correlation as per p-value reiterated the preexisting fact).

3. **Table 3: what is the significance of bold figures. e.g., in S. Typhi ceftazidine in bold with 3 resistant and not ciprofloxacin etc with 6 resistant isolates. Similar is the case with S. aureus and P. aeruginosa.**

**Response:** Thank you for the comment. This bold number(s) indicates whether each bacterial strain is statistically significant with each antibiotic. Answering the specific context of *S*. Typhi

The total number of ciprofloxacin-resistant Enterobacterales in figure 1 is much lower (n=102) than ceftazidime-resistant Enterobacterales (n=124) in figure 2. Though S. Typhi’s higher resistance to ciprofloxacin should be in significant association compared to the lower resistance rate of ceftazidime, it should be acknowledged that the 2*2 chi-square cross-tabulation performs as whole variable categories i.e., in proportion to the total, including the “other” then compared to S. Typhi. Due to the variation in total values, particularly in this context, the higher resistance incidence (total smaller) (6/102, 5.88%) in Figure 1 becomes statistically insignificant, while the lower resistance incidence (total larger) (3/124, 2.41%) in Figure 2 becomes significant.

**Figure 1. *S.* Typhi * Ciprofloxacin Crosstabulation**

**Figure 2. *S.* Typhi * Ceftazidime Crosstabulation**

**Answering the specific context of *S*. aureus**

**Figure 3.**

**Figure 4.**

Again, the exact same pattern is observed, thence, similar description covers the context for *S. aureus* and *P. aeruginosa*.

By emphasizing such significant associations in bold, the table will be curtailed, because it will have less columns per antibiotic. We have considered the comments from the reviewer as an opinion and therefore, have responded as per our insights. However, if reviewer is suggesting removing such, the authors will definitely work further on it.
