# Supplementary material for: Assessment of four in vitro phenotypic biofilm detection methods in relation to antimicrobial resistance in aerobic clinical bacterial isolates
Source: PLoS One. 2023 Nov 22;18(11):e0294646. doi: 10.1371/journal.pone.0294646 (PMC10664881; doi:10.1371/journal.pone.0294646)
Supplement: Attachment — Submitted filename: Reviewer Comments.docx [file pone.0294646.s004.docx]

The authors have well addressed the reviewer’s comments however there are minor clarifications in results required from authors.

Table 2: why no of isolates are more than no of samples of urine blood and sputum

Table 2: what is the significance of *p*-value

Table 3: what is the significance of bold figures. e.g., in *S.* Typhi ceftazidine in bold with 3 resistant and not ciprofloxacin etc with 6 resistant isolates. Similar is the case with *S. aureus* and *P. aeruginosa.*
